# Trends in road traffic accidents in Anambra State, South Eastern Nigeria: need for targeted sensitization on safe roads

**DOI:** 10.11604/pamj.supp.2019.32.1.13285

**Published:** 2019-01-25

**Authors:** Uchenna Anebonam, Chinedu Okoli, Pius Ossai, Olayinka Ilesanmi, Patrick Nguku, Peter Nsubuga, Ahmed Abubakar, Akin Oyemakinde

**Affiliations:** 1 Nigeria Field Epidemiology and Laboratory Training Programme, Abuja, Nigeria; 2 Federal Road Safety Commission of Nigeria, Abuja, Nigeria; 3 Global Public Health Solutions, Atlanta, Georgia, USA

**Keywords:** Roads traffic accidents, trends, fatal injuries, Anambra, Nigeria

## Abstract

**Introduction:**

Road traffic accidents are leading cause of injuries and deaths globally. Low income economies are the most affected. Most causes of RTA are predictable and preventable. This study describes trends and causes of road traffic accidents in Anambra State, South Eastern Nigeria.

**Methods:**

We conducted a retrospective study of road traffic accidents from 2010 to 2014. Data were obtained from the Federal Road Safety Commission, Anambra State Command. Information extracted included sex, age, cause of accidents, number of people and type of vehicles involved. Cases were recorded as fatal if any victim died, serious if any victim was hospitalized for more than 24 hours and minor if any victim was hospitalized for less than 24 hours. Causes of accidents were classified into human, mechanical, and climatic factors. Univariate analysis to generate frequencies and proportions was conducted using Microsoft Office Excel 2007.

**Results:**

A total of 1,141 road traffic accidents consisting 271 fatalities, 652 serious and 218 minor cases were recorded in Anambra State from 2010-2014. Seven thousand, four hundred and forty-four persons involving 1,816 vehicles were involved in RTA that resulted in 448 deaths and 2,785 injuries. More deaths 348 (77%) and injuries 2009 (72%) occurred more in males than females. Major causes of road traffic accidents were loss of vehicle control 256 (17%) and speed violation 207 (14%). There was an increased number of persons involved in RTA in 2014 (1,842) compared with 2010 (299). In all, 3,233 casualties (deaths and injuries) and crashes (fatal, serious and minor) were recorded out of which 900 (27.8%) casualties and 294 (9.1%) crashes occurred in 2013.

**Conclusion:**

Leading causes of road traffic accidents are human factors; speed violation, loss of vehicle control and dangerous driving which are sadly preventable. Sensitization and enforcement of safe road principles among commercial vehicles and car drivers will help curb this menace. Government at all levels should implement strong policies aimed at reducing the speed of vehicles on roads.

## Introduction

The term accident refers to any incident, occurring suddenly, unpredictably and unintentionally usually under known or unknown circumstances. A Road Traffic Accident (RTA) can be defined as an accident; usually collisions of a vehicle, with another vehicle, pedestrian, cyclist, animal, road debris, or other stationary obstruction, such as a tree or utility pole which occurs on a way or street open to public traffic. Road Traffic Accidents usually results in injury, death and property damage [1]. RTAs are a major cause of fatal and nonfatal injuries leading to permanent disability and other indirect health consequences. It is estimated that about 1.4 million people die in road crashes each year and as many as 50 million injured [2, 3]. RTAs are among the leading causes of death globally and a leading cause of death among young people aged between 15 and 29 years old [3]. In recent times worldwide, RTAs have become a major public health problem and are accorded same status as all neglected pandemics [4]. Globally, 2.1% of total deaths and 21% of total injuries have been attributed to RTA with developing and underdeveloped countries including Nigeria accounting for 80% of these deaths [3, 4]. Globally, economic, population growth and industrialization are accompanied by greater movement of people and goods with accompanying investment in transport infrastructure and this exerts a heavy pressure on the transport network [5]. Thereby leading to increase road use and contributing to RTA. The World Health Organization (WHO) Global Status Report on Road Safety for 2015 reported that the highest proportion of road fatalities for the last decade (approximately one-third of the one million global deaths) occurred in South East Asia. The year 2013 witnessed the highest number of road fatalities which was 334,000.The injury mortality rate was highest in Africa accounting for 28.3 per 100,000 population when compared with 11.0 per 100,000 population in Europe [3]. RTAs in Africa has been projected to increase from its 1990 figure of 59,000 cases to 144,000 cases annually by 2020 while developed countries will witness a decline [6].

According to Agbonkhese *et al*. (2012) the overall road traffic injury rate in Nigeria is about 41 per 1,000 population and mortality from road traffic injuries are about 1.6 per 1,000 population [7]. Recent studies conducted in Nigeria by Ukoji *et al*. (2014) from Abuja and Lagos with data from the Nigeria Watch database (an online data source) show that about 15,090 lives were lost to 3,075 RTA cases from 2006-2014. This figure has a probability of rising due to insurgency and population growth rate as reported by the authors [8]. Being mindful of the statistics on road traffic accidents in developing countries and recognizing their catastrophic effect on the economy and population, the Federal Government of Nigeria established the Federal Road Safety Commission (FRSC) in 1988 with responsibilities of policymaking, organizing and administration of road safety in Nigeria [9]. Recent studies by Agbonkhese *et al*., (2012, 2013) and Adejugbagbe *et al*., (2015) conducted in different states in Nigeria show that RTA still requires urgent attention to characterize and put in appropriate intervention from all quarters [4, 7, 9]. Routine and timely evaluation of the RTA regarding trend, the probable causes, the types of vehicles involved in the accidents, whether they are commercial or private, the number of persons involved as well as the severity of accidents is a prerequisite for the development of measures aimed at reducing the RTA in Nigeria [9]. Anambra state, a heavily industrialized and commercial State with a network of interstate and intra-city roads has recorded road traffic accidents over the years and the figure continues to rise; however, studies on the current trends and causes of RTA in the state are limited [10, 11]. We undertook this study in Anambra State to determine the pattern, identify the major causes and suggest solutions to road traffic accidents in Anambra State, South Eastern Nigeria.

## Methods

**Study area:** the survey was conducted in Awka, Anambra State, Southeast Nigeria with an estimated population of 4.2 million (projected from the 2006 National Census). Currently, Anambra State has the lowest poverty rate in Nigeria. The indigenous ethnic groups in Anambra State are the Igbo, which constitutes 98% of the population and a small population of Igala (2%) who live mainly in the northwestern part of the state. Anambra State has many other resources regarding agro-based activities like fishery and farming which plays an important role in the State’s economy. Administratively, the State has 21 Local Government Areas (LGAs) and 177 communities. Anambra State boundaries are formed by Delta State to the West, Imo State and Rivers State to the South, Enugu State to the East and Kogi State to the North. Anambra is the eighth most populated state in the Federal Republic of Nigeria and the second most densely populated State in Nigeria after Lagos State with > 60% of its people living in urbanized places.

A large modern market is located in Onitsha, which is the hub of the state’s commerce and industry. A major bridge over the Niger River at Onitsha provides a direct road link westward to Benin City and Lagos and eastwards to Port Harcourt and Owerri, with a network of roads that connect Onitsha with Awka (Anambra State Capital), Enugu and upwards to Benue State and beyond. Consequently, Onitsha has grown to become one of Nigeria´s most significant market towns and its main market, one of the largest markets in West Africa a center point where billions of Naira are transacted every day with a corresponding increase in human and vehicular movement. Nnewi one of the commercial cities in Anambra boasts of the largest collection of motorcycles in the whole of West Africa.

**Study design:** this was a cross sectional study. We reviewed secondary data of road traffic accident cases (injuries and fatalities) from 2010 to 2014 in Anambra State, Nigeria. The data were obtained from the FRSC Anambra State Command. Information extracted include sex and age, date and time of accidents, State unit where the accident occurred, probable causes of accidents, number of people that died, number and type of vehicles (or motor bikes) involved in accidents, the number of people involved in RTAs as well as categories of vehicles involved.

**Classification of terms:** accident cases were recorded and further classified as fatal if any victim died, serious if any victim was hospitalized for > 24 hours, minor if any victim was hospitalized for < 24 hours or not hospitalized at all, while casualty refers to the number of persons injured and the number of deaths in an accident case.

**Data analysis:** documentation of RTA was on Microsoft office word. The spread sheet was copied to Microsoft office excel. The data were reviewed and cleaned using Microsoft Office Excel 2007 package and we did univariate analysis to generated proportions and frequencies.

**Ethical approval:** written permission was sought and approval given by the State FRSC Sector Commander for access to data. Confidentiality of information extracted was maintained by coding and information saved in a password protected storage system.

## Results

A total of 1,141 RTAs were recorded in Anambra State from January 2010 to December 2014; 271 (23.6%) of these were recorded as fatal cases, 652 (57.1%) serious while 218 (19.1%) were minor cases. In all, the 1,141 RTAs involved 7444 persons with 48% (3,233/7,444) casualty rate, out of whom 13.9% (448/ 3,233) died and 86.1% (2,785/3,233) injured. A total of 4,211/7,444 (56.6%) persons were either unhurt or not hospitalized (Table 1). The trend in percentage change over the 5 years in review indicated a > 100% increase in all variables including the number of fatalities, the number of serious and minor cases, the number of people injured as well as the number of persons involved in RTA cases except in minor injuries, which was 40% increase 2010 compared to 2014. The percentage change in the number of persons involved in RTAs showed a 516% increase for the 5-year period reviewed (Table 1). Of the 1816 RTA cases recorded in Anambra from 2010-2014, the probable causes for 1,469 (81%) RTA cases were determined, while the probable causes for 347 (19%) were unclear. Of the 1,469 RTA cases whose causes were determined, 314 (21%) occurred in 2011, 344 (23%) in 2012, 474 (32%) in 2013 and 325 (22%) reported for 2014. The result showed a regular pattern of the increase over the 5 years reviewed with a decrease in 2014 (Table 2). The top four most probable causes of RTA from 2010-2014 in Anambra State were a loss of vehicle control which accounted for 256 (17%) of the total accident cases and speed violation which accounted for 207 (14%) of all the cases. Dangerous overtaking accounted for 184 (12.6%) of the cases while brake failure accounted for 133 (9.5) of the cases (Table 2).

**Table 1:** Trends in Road Traffic Accidents, Casualties and Number of persons involved in Anambra State, 2010-2014

	Crashes				Casualties			
Years	Fatal	Serious	Minor	All	Killed	Injured	No of Persons Involved	All
2010	16	51	30	97	39	250	299	289
2011	39	94	23	156	61	507	1110	568
2012	67	178	66	311	90	629	1947	719
2013	67	170	57	294	134	766	2246	900
2014	82	159	42	283	124	633	1842	757
Total	271	652	218	1, 141	448	2,785	7,444	3233
% Change 2010-2014	412.50	211.80	40	191.70	218	153	516	162

**Table 2:** Probable causes of road traffic accidents in Anambra State, 2010-2014

Probable causes	2010	2011	2012	2013	2014	Total	%
Loss of Control of Vehicle	4	56	103	31	62	256	17
Over speeding	3	3	64	24	113	207	14
Dangerous /Wrong Overtake	1	101	17	42	23	184	12.6
Brake Failure	1	45	34	24	29	133	9.5
Tyre Burst	1	23	9	66	13	112	7.6
Use of Phone while Driving	0	18	22	35	21	96	6.5
Weight Overload	1	5	10	52	13	81	5.5
Driving on other Vehicle route	0	29	14	21	16	80	5.5
Sleeping on steering / Fatigue	0	11	13	43	11	78	5.3
^+^Road Obstruction Violation	0	5	3	30	6	44	3
Mechanical Deficiency	0	3	16	19	4	42	2.9
Driving under Alcoholism	0	6	13	19	4	42	2.9
Sign Light Violation	0	3	9	25	3	40	2.7
Poor Weather	1	3	7	21	5	37	2.5
Bad Road	0	3	10	22	2	37	2.5
TOTAL	12	314	344	474	325	1,469	100

+ Causing Obstruction on the road

**Figure 1:**
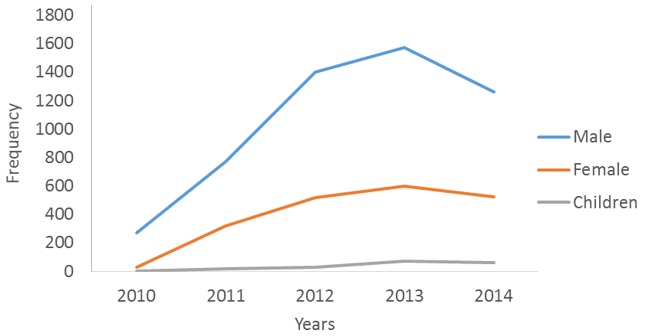
People involved in all cases of RTA in Anambra State, 2010- 2014

**Figure 2:**
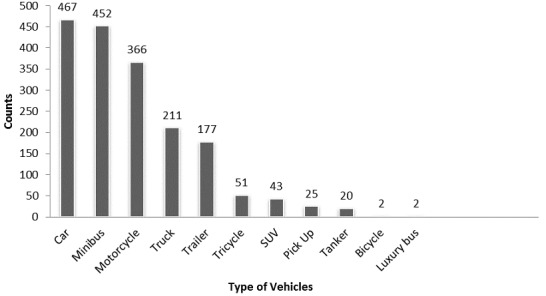
Vehicle types involved in RTA cases in Anambra State, 2010- 2014

There was a total of 7,444 individuals involved in Road Traffic Accident cases in Anambra State. 299 (4%) occurred in 2010, 1110 (15%) in 2011, 1947 (26%) in 2012, 2,246 (30%) in 2013 and 1842 (25%) in 2014 (n = 7,444). The highest number of individual involvement in Road Traffic Accident cases occurred in 2013 (Figure 1). From 2010-2014, 5,276 (71%) of the people involved in RTA cases were males, 1985 (27%) females while 183 (2%) were children under the age of 18 years as shown in Figure 1. Cars were the most common vehicles involved in RTAs in Anambra State from 2010-2014 followed by minibuses, motorcycles, trucks, trailers, tricycles, SUVs, pick-ups and tankers (Figure 2).

**Figure 3:**
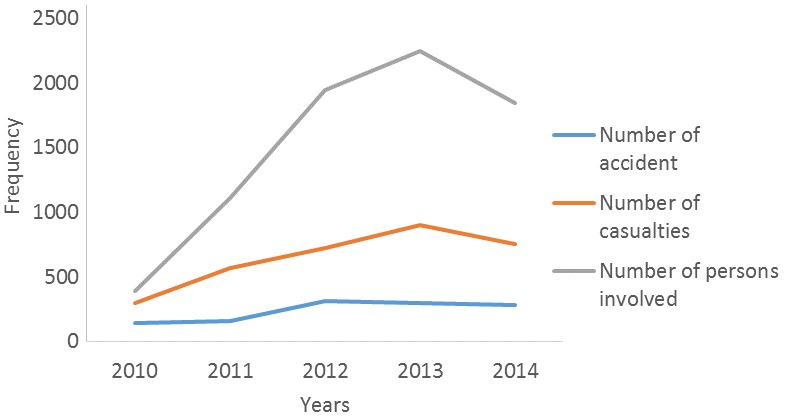
Trends of number of casualties, injuries and RTA in Anambra State, 2010-2014

**Figure 4:**
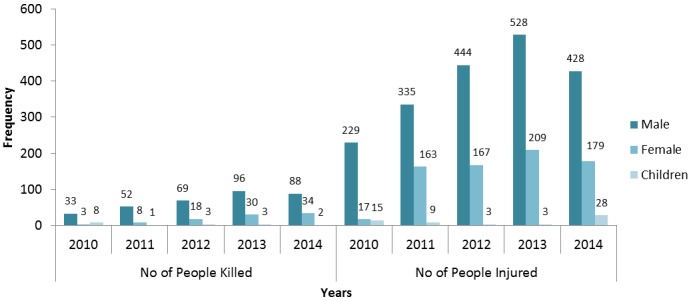
Trends in injuries and deaths in RTA cases, Anambra State, 2010-2014

The yearly trends in the RTA cases showing the number of vehicles involved, the number of casualties, the number of crashes as well as the number of persons involved is as shown in Figure 3. There were gradual increases over the years with a marked increase for all the variables in the year 2013 except in the number of crashes that remained at a plateau. The lowest number of vehicles involved in RTA in the State was recorded in the year 2011. The injury and death trends in RTA from 2010-2014 shows a gradually increased pattern over the years up to 2013 and a decline in 2014. Of the 2,785 injuries recorded over the 5 years reviewed 768 (28%) occurred in 2013 and 635 (23%) recorded in 2014. Of the 448 deaths recorded, 338 (75%) adult males were involved while 93 (21%) adult females also died. Also, 17 (4%) of the deaths were children under the age of 18 years. Yearly trends show a gradual increase in proportions from 2010 to 2013 and a decline in 2014 for all parameters; the number of males, females and children involved in RTA (Figure 4).

## Discussion

We undertook this study in Anambra State to determine the pattern of RTA, identify the major causes and suggest solutions. Our review of the trends of RTA in Anambra State from January 2010-December 2014 revealed a consistent increase in the number of road crashes, deaths as well as injury rates, the number of people involved as well as the number of vehicles involved with a noticeable spike in 2013 and a gradual decline in 2014. The marked increase in RTA cases may be attributed to the increase in both vehicles and people in 2013 occasioned by the massive relocation of displaced persons from the northern part of the country to the south east due to the insecurity in the north prompted by the insurgency. Our finding is similar to findings from a study conducted in Abuja by Ukoji *et al*., in 2014 which found that an upsurge in the human and vehicular movement has resulted in more fatal road traffic accidents [8]. Similar studies in India, Kenya and Ethiopia have reported this effect of upsurge on RTA [1, 5, 12]. The steady decline observed from 2014 could be adduced to return to normalcy and less vehicular and human movement.

Human factors (e.g., speed violation, loss of vehicle control and dangerous overtaking) were the leading causes of road traffic accidents from 2010-2014. This finding is similar to previous studies conducted on RTAs in Owerri, Imo state, Nigeria by Ohakwe *et al*. and Ibadan by Adejugbagbe *et al*. [4, 9]. The finding further supports the WHO Global status report on road safety, 2015 which reported that leading causes of road traffic accidents are both predictable and preventable and that changing user behavior is a critical component of the holistic “safe systems” approach [3].

There was a preponderance of cars, minibuses and motorcycles involved in RTA in Anambra. While it is not surprising that cars and mini buses were more involved in RTA than other vehicles, as many studies have highlighted this trend over the years in different climes [3, 12-15]; it is, however, worrisome that motorcycle constituted 20% to the total RTA considering the limited safety of occupants on impact. This high rate of motorcycle accidents has been reported previously [10, 14]. Injuries and deaths occurred more among males than females; this figure has been reported [4, 6-7, 12, 14]. Men are more involved in travelling than women, since meeting the financial needs of the family rest on them often. In carrying out this secondary data analysis, the limitation encountered was the use of only data obtainable from the FRSC. Few accident cases not captured by the FRSC could have gone unreported.

## Conclusion

The findings from our study have further shown that road traffic accidents remain a growing pandemic. It has also further shown the priority targets for prevention and health education; motorcyclists and car drivers. We further recommend training and orientation of drivers on road signs and rules before issuance of driving licenses. The introduction of bumps on major roads to limit speed should be prioritized.

### What is known about this topic

- RTAs are a major cause of fatal and nonfatal injuries leading to permanent disability and other indirect health consequences;
- RTA still requires attention by all road users;
- Pattern of RTA is poorly characterize in Nigeria.

### What this study adds

- Road traffic accidents has become a growing pandemic;
- The trends and causes of RTA in Anambra State were described;
- Priority targets for prevention and health education were identified.

## Competing interests

The authors declare no competing interests.
